# Cookies Fortified with *Clitoria ternatea* Butterfly Pea Flower Petals: Antioxidant Capacity, Nutritional Composition, and Sensory Profile

**DOI:** 10.3390/foods13182924

**Published:** 2024-09-15

**Authors:** Ribi Ramadanti Multisona, Kamila Myszka, Bartosz Kulczyński, Marcellus Arnold, Anna Brzozowska, Anna Gramza-Michałowska

**Affiliations:** 1 Department of Gastronomy Science and Functional Foods, Faculty of Food Science and Nutrition, Poznań University of Life Sciences, Wojska Polskiego 31, 60-624 Poznań, Poland; ramadanti.multisona@up.poznan.pl (R.R.M.); bartosz.kulczynski@up.poznan.pl (B.K.); marcellus.arnold@up.poznan.pl (M.A.); anna.brzozowska@up.poznan.pl (A.B.); 2 Department of Biotechnology and Food Microbiology, Faculty of Food Science and Nutrition, Poznań University of Life Sciences, Wojska Polskiego 48, 60-627 Poznań, Poland; kamila.myszka@up.poznan.pl

**Keywords:** *Clitoria ternatea*, functional food, antioxidant, cookies, apple peel, oxidation

## Abstract

This study aimed to fortify cookies to be functional food by adding *Clitoria ternatea* flower (CT) at concentrations ranging from 0 to 8%. Sensory profiling identified 6% CT as optimal for organoleptic attributes. The addition of CT did not significantly impact protein, lipid, and ash content but decreased energy value and increased insoluble and soluble fibre levels. The inclusion of 6% CT had a significant effect on the overall total phenolic content (TPC), which increased compared to the control sample. Antioxidative activity analyses showed enhanced antioxidative activity in ABTS, DPPH, ORACFL, and PCL assays. The addition of 6% CT inhibited hydroperoxide production in cookies. However, over a period of 6 weeks, a significant rise in peroxide value was observed during the 4th and 6th weeks of storing fortified cookies. All assessed products met the high microbiological quality standards. The sensory evaluation scores showed that CT can create cookies with health benefits and a good overall acceptance score. The texture of the cookies gradually became softer, but no significant changes in visual appearance were observed. CT can be extensively used in baked cookies as a rich source of polyphenols with strong antioxidant properties and high fibre content, as well as a fortification source for the development of functional foods.

## 1. Introduction

Growing awareness of the importance of consuming healthful foods has resulted in a surge in the demand for functional foods. Studies have demonstrated that the beneficial effects of functional food on health are attributed to the presence of bioactive compounds, including polyphenols, dietary fibre, vitamins, probiotics, prebiotics, fatty acids, and minerals [1,2,3,4]. Recently, there has been an increase in interest from the food industry in the development, evaluation, and marketing of novel foods. Particular emphasis has been placed on reduced manufacturing costs and whole foods, which can frequently be a source of biologically active components [1]. The efficacy of functional food in enhancing health will diminish if it is not consumed on a regular basis [5]. Simply stated, it must be a component of a dietary regimen that is recognised for promoting good health and overall well-being. Cookies, along with other confectionery products, are extremely popular as sweet treats. Due to its high nutritional content, long shelf life, variety of tastes, affordability, and popularity, cookies are a valuable alternative for dietary supplementation, which is desired by consumers [6].

Researchers started applying natural antioxidants to preserve the nutritional quality of food, inhibit the formation of harmful oxidation products, decrease rancidity, and prolong the shelf life of food products [7,8]. The food industry is increasingly embracing natural antioxidant substances such as total phenolic and total anthocyanin compounds because of their various health benefits, which include anti-inflammatory effects, antioxidant effects on human low-density lipoproteins, improvement in eye function, antidiabetic and protection against cancer and cardiovascular diseases [9]. Thus, the incorporation of antioxidant-rich fruits, vegetables, and plants in cookies is an innovative approach to creating functional foods and enhancing the quality of food items.

*Clitoria ternatea* (CT), commonly referred to as butterfly pea or blue pea, is a plant species classified under the Fabaceae family. CT is widely recognised as a prominent source of polyphenols, which possess a potent antioxidant capacity. The analysis of CT flower components revealed the existence of polyphenols as secondary metabolites, including tannins, phlobatannin, phenols, flavanoids, flavonol glycosides, and anthocyanins [10]. A previous study by Manjula et al. [11] qualitatively identified the existence of phytochemicals, including alkaloids, tannins, glycosides, resins, steroids, saponins, flavonoids, and phenols. The phenolic content in CT flower has been found to have a strong correlation with its antioxidant activity, which in turn has pharmacological effects and benefits for human health. These benefits include antidiabetic, anticancer, antimicrobial, and anti-inflammatory properties [10,12,13]. The flower petals of CT contain high levels of anthocyanin pigments, making them a promising natural source of colourants for various meals and beverages [13,14]. The CT flower not only provides aesthetically pleasing colours to food products, but it also exhibits a diverse range of pharmacological qualities that contribute to health-promoting effects [15,16]. In addition to their phytochemical properties, the nutritional profile of CT has been documented by Neda et al. [17]. The flowers contain 2.5% fat, 2.2% carbohydrates, 2.1% fibre, and 0.32% protein, with a moisture content of 92.4%. They are also notably rich in minerals, including calcium (3.09 mg/g), magnesium (2.23 mg/g), potassium (1.25 mg/g), zinc (0.59 mg/g), sodium (0.14 mg/g), and iron (0.14 mg/g).

The food industry produces an enormous amount of waste each year, which can be used as a valuable source of high-quality ingredients like proteins, fibres, polysaccharides, antioxidants, and aromatic compounds. These ingredients can be used in the development of new foods, pharmaceuticals, cosmetics, and other products [18,19]. Apple peel, which accounts for a significant amount of industrial waste, can be developed into high-value by-products [20]. The peel is higher in lipids, ash, soluble dietary fibre, flavonoids, and phenolic acids than the pomace [20,21]. Apple peels contain a higher concentration of phenolic compounds than other edible sections of the apple. The flesh of an apple contains catechins, procyanidins, phloridzin, phloretin glycosides, caffeic acid, and chlorogenic acid; the peel contains all of these compounds as well as additional flavonoids not found in the flesh, such as quercetin glycosides [22]. Dietary fibre and phenols both assist in preventing diseases and improving human health [23,24].

The present literature discussed extensive research on food fortified with various extracts derived from CT flowers. However, there is a lack of studies exploring the use of dried petals [13,16]. There is currently a shortage of butterfly pea-fortified items in the marketplace, leading to the idea of developing similar products. Developing cookies fortified with dried CT flower petals would meet the growing demand for food that promotes health and offers high nutritional value. Incorporating food processing by-products such as apple peel into this product development can also be an effective way to add value, reduce waste, and create innovative products. Developing food products with unique and appealing characteristics would address the existing demand in the food market. Consequently, the study was conducted to examine the nutritional content and antioxidant properties of cookies fortified with dried CT flower petals. The aim was to confirm the extension of the product’s shelf life and the potential health advantages it offers to health-conscious individuals seeking to enhance their daily antioxidant intake.

## 2. Materials and Methods

### 2.1. Research Design

The research was conducted on cookies with the addition of dried CT flower petals (Dary Natury Sp. z o. o, Grodzisk, Poland, Eco-guarantee certification body PTRE sp. z o.o.) and a control sample with no additives. The cookies were assessed based on their basic composition, antioxidative activity, and sensory evaluations over a 6-week period of storage at an ambient temperature (±20 °C).

### 2.2. Preparation of Cookies

The dried CT flower petals fortified cookies were produced according to Table 1. To initiate the process, the dried flower petals from CT were pulverised into a powder. The apple peels were dried using a freeze dryer and subsequently pulverised into a fine powder. The initial stage of cookie production involved combining coconut oil, butter, and sweetened condensed milk. Next, the flour, salt, apple peel powder, and dried CT flower powder were combined and thoroughly blended until a smooth consistency was achieved. Subsequently, the dough was moulded to a uniform thickness of 7 mm and then divided into round forms using a cookie cutter with a diameter of 3 cm. The moulded dough was thereafter positioned on the baking tray and subjected to a temperature of 140 °C for a duration of 25 min.

### 2.3. Chemical Composition

The CT flower-fortified cookies’ chemical composition comprised fat, protein, ash, carbohydrate, and fibre. The lipid content was assessed using the Soxhlet method [25] (Soxtec System, Foss Tecator). The protein content was evaluated using the Kjeldahl method [26] (Foss Tecator). The ash content was evaluated after the sample had been completely burnt in an oven [27]. Carbohydrate content made up the difference between 100 and the sum of protein, fat, water, and ash. The fibre fractions were assessed as total (TDF), insoluble (IDF), and soluble (SDF) dietary fibre content using the enzymatic–gravimetric Asp technique [28]. Total phenolic content (TPC) was determined using the assay presented by Shahidi and Naczk [29], based on the reduction in Folin–Ciocalteu reagent coloured complexes at λ = 725 nm (SP-830 plus). TPC contents were evaluated on the basis of a standard curve for y = 4.4721x − 0.0313 (R^2^ = 0.9968) and expressed as mg of gallic acid equivalents (GAE)/100 g of product.

### 2.4. Antioxidative Potential Analysis

The cookies were evaluated for their antioxidant activity during the storage, and the samples were stored in a thermostat (20 °C) in a dark condition for 2 months. The duration of storage was determined based on the estimated period during which the consumer can keep the product on a shelf without any degradation in quality. Before conducting the antioxidant activity analysis, the samples were ground and extracted. The cookies samples were assessed for their antioxidative activity utilising the ABTS, DPPH, and ORAC_FL_ assays. The ABTS radical cation scavenging activity was tested using spectrophotometric measurement (λ = 734 nm) by Re et al. [30] to determine their antioxidant ability to scavenge blue-green ABTS generated from potassium persulfate oxidation. Antioxidants reduce ABTS cation radical intensity [31]. The ABTS radical cation scavenging percentage was calculated using the standard curve y= 187.41x + 1.5483 (R^2^ = 0.998). The product has mg of Trolox equivalent (TE) per 100 g. The DPPH assay was conducted using the Sánchez-Moreno et al. [32] method, which measures DPPH solution absorbance decrease at λ = 515 nm in the presence of antioxidants. The DPPH radical scavenging percentage was calculated as mg TE per 100 g of product using the standard curve equation y= 91.464x − 6.555 (R^2^ = 0.9945). Previous research (Gramza-Michałowska and Korczak) [33] assessed the potential of fluorescein to scavenge peroxyl radicals using the ORACFL test. The F-2700 fluorescence spectrophotometer (Hitachi, Tokyo, Japan) was used to measure fluorescence at 493 nm excitation and 515 nm emission. Data were then analysed using a standard curve y = 0.1728x + 0.0364 (R^2^ = 0.9995) and given as mg TE per 100 g product. The PCL assay was conducted according to the methodology described by Besco et al. [34]. The PCL assay relies on the inhibition of luminol’s photo-induced autooxidation, which produces blue-glowing chemiluminescence, by antioxidants. This inhibition is mediated by the radical anion superoxide (O2•-) [34]. The outcome was expressed as mgTE per 100 g of the product.

### 2.5. Lipid Peroxide Value Analysis

Prior to the determination, lipid extraction was performed. The lipid content of the crushed cookies was extracted using a combination of chloroform and methanol (in a ratio of 1:1.5, volume to volume), with the addition of water. The mixture was agitated for a duration of 1 h and subsequently filtered to separate the lipid fraction from the precipitate. Subsequently, the separated part was subjected to evaporation at ambient temperature in order to acquire a refined lipid fraction over the course of one night.

In order to determine the peroxide value, the lipid fraction was combined with a starch solution in an acidic environment. The solution, which had a dark blue colour, was subjected to titration using sodium thiosulfate until it became colourless. Results were expressed in meq O_2_/kg [35]. PV was calculated according to the following formula:

$$Pv=\frac{\left(V-V_{0}\right)\cdot c\cdot F}{m}\cdot 1000$$

where V equals to the volume of titrate used for sample titration, V_0_ is the volume of titrate used for blank titration, c is the concentration of sodium thiosulfate used (mol/L), and F is the exact concentration of sodium thiosulfate.

### 2.6. Microbiological Identification

The preparation of samples for microbiological determinations followed the recommendations of the ISO standard [36]. Briefly, samples, after weighing on an analytical scale, were suspended in a sterile saline solution at a mass ratio of 1:10. Samples were then mechanically homogenised in a Pulsifier (Microgen, London, England). Each sample was homogenised in a separate stomacher bag (Merck, Darmstadt, Germany). The starting suspensions were diluted using the decimal dilution method. Samples were inoculated in duplicate into Petri dishes, which were inoculated with appropriate microbiological media: PCA agar, PDA agar, VRBG, and Baird-Parker (all BTL media, Łódż, Poland). In the procedure for the identification of aerobic mesophilic microorganisms, *Enterobacteriaceae* and *Staphylococcus aureus*, incubation of the samples was carried out at 30 °C for 48 h [36,37,38,39]. In the psychrophilic/psychrotrophic bacteria detection procedure, incubation of the samples was carried out at 15 °C for 72 h [40]. In the *Salmonella* sp. identification procedure, 25 g of product was preincubated in 225 mL buffered peptone aqueous solution (Oxoid, UK) for 20 h at 30 °C [41]. Samples were then streaked onto selective media, RVS, and MKTTn (all BTL, Poland). The incubation process was continued at 30 °C for 24 h. For the differentiation of microorganisms, media, XLD (Merck, Germany) and BGA (BTL, Poland) were used.

### 2.7. Sensory Evaluations

A sensory analysis was conducted on freshly baked cookies and cookies that had been stored for 2–6 weeks. The analysis was carried out by a panel of 15 consumer panellists. The sensory analysis employed the sensory profiling method as described by Gramza-Michałowska et al. [1] with some modifications. The panellists assessed the samples in sensory profiling, considering their appearance, smell, texture, and taste. The evaluation scale ranged from 0 to 9, representing the absence of extremely high/very intensive, using the methodology of Bagaud et al. [42] with a few modifications. The sensory analysis was carried out in a dedicated sensory analysis room, where all the consumer panellists underwent training for the testing technique. The study did not require ethical approval. Participants were informed of the purpose of the study and that their participation was entirely voluntary; therefore, they could stop the analysis at any time, and their responses were anonymous. The authors did not ask for sensitive data or personal information. Formal reliance was not used to recruit participants for the study. The mean, variance, and standard deviation were computed for each attribute of every sample and session individually.

### 2.8. Colour Measurement

The colour measurement was performed according to the method described by Liew et al. [43] and carried out using the Spectrophotometer CM-5. The specimens were placed in the Petri dish, after which the measurements were taken. The measurement and expression of colour intensity were conducted using the CIE L*, a*, b* coordinate system. The symbol L* denotes the attribute of lightness, a* denotes the attribute of red (positive value) or green (negative value) colour, and b* denotes the attribute of yellow (positive value) or blue (negative value) colour.

### 2.9. Statistical Analysis

The experiments were carried out in triplicates. The study employed one-way ANOVA and *t*-test to assess the differences in means among the samples. To further determine these differences, Tukey’s multiple range test was utilised with a significance level of *p* < 0.05. The Pearson correlation coefficient was calculated to evaluate the relationship between the total phenolic content of each treatment of cookies and their antioxidative actions, as well as lipid peroxide value. Data analysis was conducted using SPSS 20.0 (SPSS for Windows, SPSS Inc., Chicago, IL, USA).

## 3. Results and Discussion

### 3.1. Sensory Profiling of Fresh Samples

**Figure 1:**
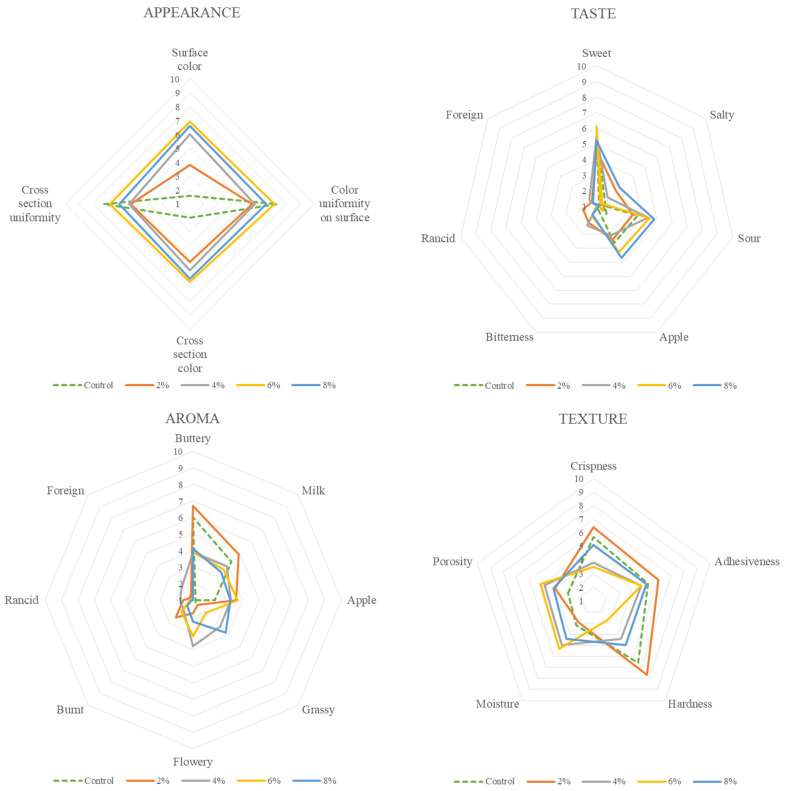
Radar plots of sensory evaluation of fresh cookies in different concentrations of CT.

A sensory profile study was performed to determine which formulation possesses the most desirable features and can be considered acceptable. For the optimisation procedure, CT samples were selected at concentrations of 0, 2, 4, 6, and 8% (Figure 1). In general, cookies containing 6% CT flower exhibit the lowest scores in undesired attributes, suggesting that this formulation is the most suitable for further analyses.

**Figure 2:**
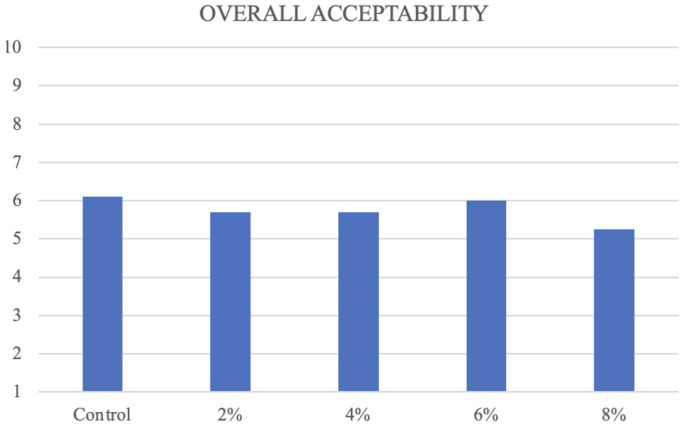
Overall acceptability of organoleptic characteristics of fresh cookies in different concentrations of CT.

According to visual outcomes for the appearance of cookies, it is noticeable that the differences occurred solely in the colour of the surface and cross-section, which can be attributed to varying concentrations of CT. The panellists observed that the surface and cross-section showed similar uniformity. The sample containing 6% CT exhibits the lowest score in unfavourable aroma characteristics, including grassy, rancid, and foreign aromas. Among the various samples, cookies containing 6% CT flower had the lowest scores in terms of bitterness, rancidity, and foreign taste in taste sensory evaluation. When it comes to texture, both adhesiveness and hardness play a significant role. When compared to other samples, cookies containing 6% CT exhibit the least adhesiveness and hardness. Furthermore, when compared to other concentrations, cookies containing 6% CT exhibit the highest level of overall acceptance (Figure 2), which is nearly identical to the score of the control sample in comparison with other concentrations.

Therefore, cookies containing 6% CT flower were selected for further research with the control sample.

### 3.2. Colour Measurement

Table 2 presents the impact of different concentrations of CT flower addition on the colour attributes of cookies. The results were represented by Hunter L*, a*, and b* values, which corresponded to lightness, redness, and yellowness, respectively.

There are significant differences on the surface of the cookies between the control sample and the samples with CT flower addition (*p* < 0.05). The control cookies had higher L*, a*, and b* values for their surface colour compared to the cakes substituted with CT flower. This indicates that the control cookies were lighter, more reddish, and more yellowish than any of the cookies containing CT flower.

**Figure 3:**
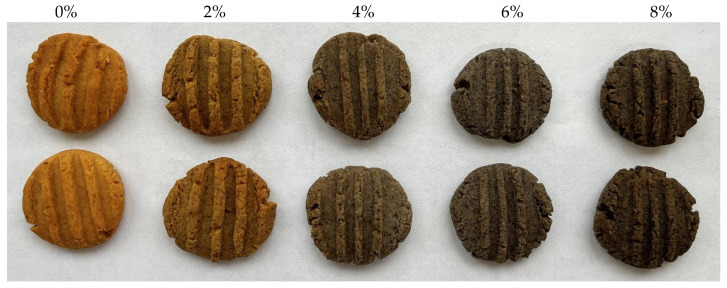
The appearance of cookies control, 2%, 4%, 6%, and 8% of CT flower.

Increasing the percentage of CT flower resulted in a decrease in the L*, a*, and b* values, indicating that the cookies became darker in colour, with reduced redness and increased greenness, as well as reduced yellowness and increased blueness. Consequently, the presence of CT flowers had a significant impact on the colour of the cookies’ surface. The findings align with a previous investigation conducted by Pasukamonset et al. [44] on sponge cake made using CT extract. The study stated that the presence of substituted ingredients and their interactions contributed to the production of darker baked cakes. Figure 3 displays the visual representation of cookies that include CT flower.

To conduct a comprehensive analysis, the E index was devised to quantify the colour change by combining the L*, a*, and b* values. The E index was determined using the equation E = ((L*)^2^ + (a*)^2^ + (b*)^2^)^1/2^, with the greatest influence coming from the lightness [45]. The cookies containing CT flower exhibited lower E values compared to the control cookies. The results also indicated an inverse relationship between the trend in E values and the amount of flower used in the cakes, suggesting that the brightness of the cakes decreased due to the presence of CT flower [44,46]. Thus, it is possible that the purple pigments in the CT flower are accountable for the alterations in colour observed in cookies supplemented with this flower.

### 3.3. Chemical Composition

The basic chemical composition of freshly baked cookies was assessed, both with and without the addition of CT. The analysis findings are displayed in Table 3. The energy content of control cookies and cookies containing 6% CT is 513.38 and 509.90 kcal/100 g, respectively. The energy levels were comparable to those reported in a recent study on butter cookies with floral substitutions [47] and a commercially available butter cookie brand, Krakuski (Poland). Nevertheless, it is crucial to note that the use of apple peel powder and dried CT flower in this research augmented the antioxidant and fibre levels of the cookies, rendering them more nutritious and functional compared to the commercially available alternatives. The use of 6% dried CT flower had no significant impact on the protein, carbohydrate, fat, and ash content (*p* > 0.05). In a comparable way, Thanh et al. [48] conducted a study that found that incorporating CT into a bread product did not have a significant impact when compared to the control group.

The amounts of dietary fibre and its fractions found in the samples of butter cookies are displayed in Table 4. The cookies containing 6% CT flower had significantly greater levels of insoluble dietary fibre (IDF) and soluble dietary fibre (SDF) compared to the control group (*p* < 0.05). The presence of a significant amount of fibre in dried CT, specifically 27.63 g/100 g of dried flower, as reported by Neda et al. [17], may be the origin of this phenomenon. In a prior study conducted by Khumkhom [47], it was shown that adding flowers to butter cookies resulted in a considerable increase in fibre content. Studies by Arnold et al. [4] and Brummer et al. [49] have established a correlation between soluble dietary fibre and reductions in blood sugar and cholesterol levels. Additionally, it has the capacity to serve as a prebiotic by acting as a nourishment for intestinal microorganisms. This might result in a decrease in the pH of the colon during the process of fermentation and enhance the absorption of vital minerals [3]. However, insoluble dietary fibre mostly adds weight and promotes the movement of the intestines, even though it may only undergo partial fermentation in the colon [28]. The cookies with 6% CT flower had a considerably greater total dietary fibre (TDF) compared to the control sample (*p* < 0.05). The cookies can be classified as a high-fibre food. As to Regulation No. 1924/2006 by the European Parliament and Council, food that has a minimum of 6 g of fibre per 100 g can be labelled as high in fibre.

### 3.4. Total Phenolic Content and Antioxidative Potential

**Figure 4:**
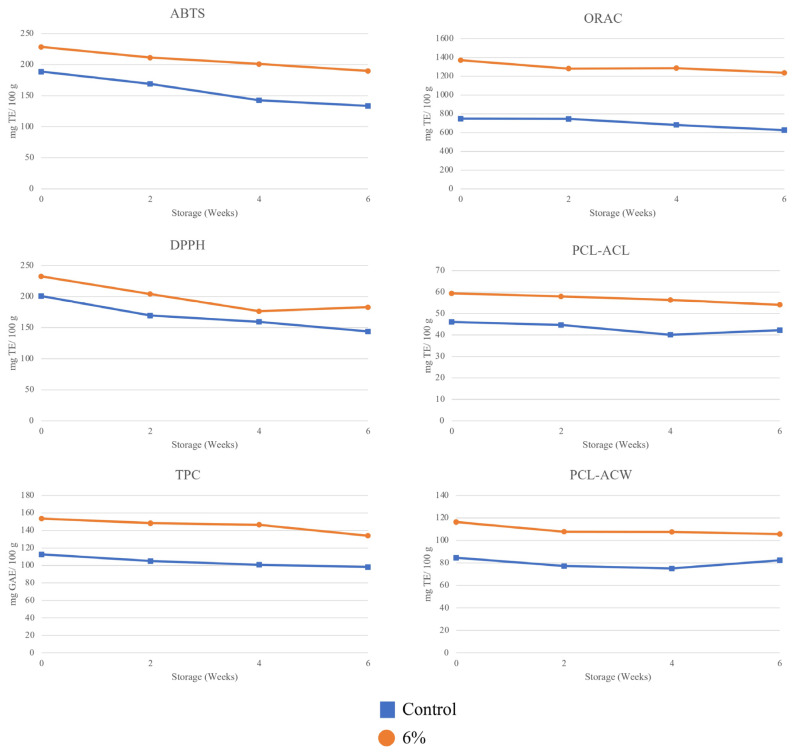
Total phenolics content and antioxidative activity with CT.

The analysis findings of the total phenolic content (TPC) and antioxidative activity of cookies, which were made and stored for six weeks, were obtained using multiple methods (ABTS, DPPH, ORAC, PCL tests). These results are presented in Figure 4. The addition of 6% CT flower to the cookies had a significant effect on the overall phenolic content, as observed in the antioxidative activity testing. The findings align with prior research indicating that incorporating CT flower into bakery items, such as sponge cake [44], cupcakes [48], and Chinese steam bread [50], resulted in elevated levels of total phenolic content and enhanced radical scavenging activity compared to the control sample without treatment.

In general, the total phenolic content and antioxidant activity decrease as the storage time increases. Over a period of 6 weeks, there was a notable decrease in total phenolic content (TPC) in both the control cookies (from 112.76 to 98.31 mg GAE/100 g) and the CT flower extract (from 153.42 to 134.08 mg GAE/100 g) (*p* < 0.05). Nevertheless, it is evident that there is no significant difference between both of the samples after 2 and 4 weeks of storage. The ABTS radical cation scavenging activity exhibited a significant decrease (*p* < 0.05), with a reduction of approximately 29% in the control sample and 16% in the sample containing 6% CT flower. The capacity of cookies to remove the DPPH radical decreased significantly (*p* < 0.05) in the control sample (from 200.9 to 144.02 mg TE/100 g) and in the sample containing 6% CT flower (from 232.61 to 183.04 mg TE/100 g). Contrarily, the DPPH assay indicates a notable difference in the outcome, as it demonstrates a rise in the antioxidant activity of cookies containing 6% CT flower after 6 weeks of storage.

Various in vitro assays employed to quantify antioxidative activity have diverse outcomes. The ABTS cation radical and the DPPH radical, which are radical or oxidant molecules, enable antioxidant chemicals to undergo distinct reactions [51]. A study found that different antioxidants have varying IC50 values. It also mentioned that when the wavelength used to measure changes in radical concentration does not match the spectrum of the antioxidant or biological system being analysed, ABTS and DPPH assays are suitable for determining antioxidative activity. Alternatively, the difference between the real and spectrophotometrically projected IC50 values is dependent upon both the quantity of residue in the measurement device and the extinction coefficient of the measured wavelength. Anthocyanins, found in high amounts in CT flower [13], have a strong absorption at a wavelength of 500–550 nm, which is similar to the wavelength used in the DPPH assay (515–528 nm). As a result, they can potentially influence the results and interpretation of antioxidative activity [31].

The ORACFL assay, commonly employed to determine the antioxidant capacity indicated on food labels, yielded similar outcomes. It revealed a significant decline in antioxidant levels after 6 weeks of storage (*p* < 0.05). Nevertheless, it is evident that there is no significant difference between the fresh samples and those held for two weeks in both cases. All of those results aligned with the total phenolic content, which indicated a decrease in total phenolic content in longer storage time. The samples’ antioxidants effectively eliminated free radicals, which are responsible for various oxidation events, during storage. For example, the hydroxyl groups of phenolics were utilised to give a proton or an electron. The scavenging activity diminishes as the storage period increases due to the reduction in free radicals [52]. During a 3-month storage period, cookies enriched with 5.5% ground green tea leaves exhibited a similar decrease in antioxidant activity, as indicated by ABTS and DPPH tests [1]. Similarly, gingerbread supplemented with chicken eggshell calcium exhibited a consistent pattern in ABTS, DPPH, and ORAC assays over a 2-month storage period, as observed by Arnold et al. [4].

The photochemiluminescence assays (PCL) are efficient, simple, and repeatable, making them a valuable biomonitoring tool, especially for food technology and nutrition applications [34]. The PCL assay was employed to evaluate the antioxidant capacity of the lipid-soluble fraction (ACL) and the water-soluble fraction (ACW). Hydrophilic antioxidants typically engage with oxidants in both blood plasma and cytoplasm, whereas hydrophobic antioxidants safeguard cell membranes from lipid peroxidation. In general, there was a lowering trend observed in the results of the PCL assay for both ACL and ACW (Figure 4), independent of their significance, in both the control and treated samples. However, there is an increase in scavenging activity observed at 6 weeks of storage in the control cookies in both the ACL and ACW experiments. Gramza-Michałowska et al. [1] reported a comparable occurrence in cookies that were fortified with ground green and yellow tea, as well as in the control cookies, after one month of storage. In addition, gingerbread supplemented with chicken eggshell calcium exhibited an elevated level of antioxidative activity following 1 month and 2 months of storage [4].

Overall, the total phenolic content and antioxidant activity were significantly different at each storage time and decreased as the storage time increased. The anthocyanins derived from *Clitoria ternatea* exhibit high antioxidant activity, especially at neutral to slightly acidic pH levels (pH 4–7). This antioxidant capacity helps in reducing oxidative stress in food products, thereby prolonging their shelf life and maintaining quality during storage [53]. Therefore, the incorporation of *Clitoria ternatea* anthocyanins into food products not only enhances their antioxidant capacity but can also serve as a natural preservative [13]. This dual function can improve the overall quality and safety of food products during storage, making them more appealing to health-conscious consumers.

The statistical analysis of Pearson’s correlation coefficient (Table 5) was used to assess the relationship between the TPC and antioxidative activities of the cookie samples. The total phenolic content (TPC) showed a good correlation with the antioxidative activities measured by ABTS, DPPH, ORAC, PCL-ACW, and PCL-ACL in both samples of cookies. However, there was a disparity in the correlation between TPC and PCL-ACW in control cookies, as it was not significantly correlated. Based on the PCL-ACW result trend, there is a rise in antioxidative activity during the 6-week storage period, which could potentially weaken the correlation. This finding lines up with a recent study where the authors observed positive but somewhat weak correlations (r-value of 0.417) between the total phenolic content (TPC) and antioxidant capacity (ACW) of several Thai drinks [54]. Various compounds in CT and apple peels contributed to the antioxidant activity and total phenolic content results of the cookies. The antioxidant activity can be influenced by the presence of other bioactive compounds, leading to complex interactions that may affect the strength of the correlation [55]. For example, the synergistic effects among different phenolic compounds can complicate the determination of their individual contributions to antioxidant capacity.

Cookies with 6% CT have the potential to be a promising functional food due to their strong antioxidant activity. The inclusion of apple peel powder as a substitute for flour in cookies resulted in an enhancement of their antioxidant properties, as demonstrated in a previous study conducted by Nakov et al. [21]. In addition, prior research has indicated that incorporating CT into food products, such as Chinese steam bread [50], water kefir [56], and sponge cakes [44], enhances their antioxidative properties. Therefore, the inclusion of apple peel powder in both the control and treated cookies, as well as the addition of 6% CT flower to the cookies, enhances the antioxidant activity.

### 3.5. Lipid Peroxide Value Analysis

Fat is a crucial ingredient in cookies. The product experiences oxidation of the unsaturated fatty acids, resulting in an undesirable outcome. The oxidative stability of the product is influenced by the pro/antioxidant properties of flour and other ingredients. To obtain accurate information regarding the stability of shelf life, it is recommended to analyse both primary and secondary oxidation products simultaneously [57]. Lipid oxidation is a common occurrence in cakes that have a higher lipid content. Lipid hydrolytic degradation is a significant factor leading to oxidative instability in bakery goods that are baked at high temperatures [58]. The lipid peroxide value of both the control and treated cookies was assessed at various storage times (as shown in Table 6) and represented in milliequivalents of oxygen per kilogram (meq O_2_/kg). The peroxide value is an indicator of the oxidative condition of the tested oil, with a lower peroxide value indicating higher oil quality.

By comparing the control cookies with those containing 6% CT flower, the addition of 6% CT flower to the cookies had a significant impact on the lipid peroxide value (*p* < 0.05), which was lower than that of the control sample. This finding aligns with a prior investigation on sponge cakes enriched with CT flower extract, which showed that the inclusion of the extract leads to a notable decrease in the peroxide value of the cakes [44]. The phenolic compounds and their antioxidative action contribute to the distinct reduction in lipid oxidation.

Over a period of 6 weeks, the level of lipid peroxides showed a substantial increase in both the control and treated samples (*p* < 0.05). There is no notable disparity in the treated sample during the storage periods of 2 and 4 weeks when compared to the fresh sample. Therefore, the inclusion of 6% CT flower can effectively decrease lipid oxidation during storage. This finding is consistent with the study conducted by Gramza-Michałowska et al. [1], which showed that the inclusion of green tea leaves, known for their high antioxidant content, in cookies considerably suppressed the degree of lipid oxidation during storage, as compared to the control group. This discovery aligns with the findings of Pasukamonset et al. [44], who suggested that the decrease in lipid peroxide levels in cakes could be attributed to the abundant presence of phenolic compounds in CT flowers, which function as antioxidants by scavenging free radicals. The relationship between TPC and lipid peroxide values is shown in Table 5, demonstrating a substantial association between TPC and lipid peroxide values. The correlation between the TPC and lipid peroxide value of cookies is negative, meaning that as the TPC increases, the lipid peroxide value decreases. This finding aligns with a previous study that showed a strong association between the amounts of phenolic compounds in the extract from CT and the ability to inhibit lipid oxidation in a cooked pork patty model [46]. Based on these findings, the decrease in lipid peroxide value in cookies containing 6% CT flower, especially during storage, can be attributed to the high phenolic content and strong antioxidative activity of the CT flower.

### 3.6. Microbiological Identification

All assessed products met the high microbiological quality standards (Table 7). No food saprophytic microorganisms, i.e., mesophilic/psychrotrophic bacteria, yeasts, and moulds, were found in any of the samples. The production of cookies was carried out under high sanitary regimes, which is also evidenced by the absence of *Enterobacteriaceae* bacteria in the tested material. No pathogenic microorganisms of the genera *Staphylococcus* and *Salmonella* were confirmed.

Besides the high sanitary standards during production, CT flower and apple peels contain various bioactive compounds such as phenolics, flavonoids, and antioxidants that exhibit antimicrobial properties [16,22]. Previous studies have reported that apple peel extracts demonstrated high antibacterial activity against pathogenic bacteria such as *Listeria monocytogenes*, *Staphylococcus aureus*, *Escherichia coli*, and *Salmonella* spp. [59,60]. Moreover, one study also reported that the addition of CT flower to muffin dough provided extended shelf life as well as inhibitory activity against foodborne bacteria, including Gram-positive bacteria like *Escherichia coli, Proteus mirabilis, Bacillus cereus*, and *Staphylococcus aureus*, as well as Gram-negative bacteria like *Yesirnia* sp., *Pseudomonas aeruginosa*, and *Bacillus coagulans* [61]. Therefore, the inclusion of CT flower and apple peel in the cookie formulation also contributed to the absence of unwanted microorganisms.

### 3.7. Sensory Profiling during Storage

**Figure 5:**
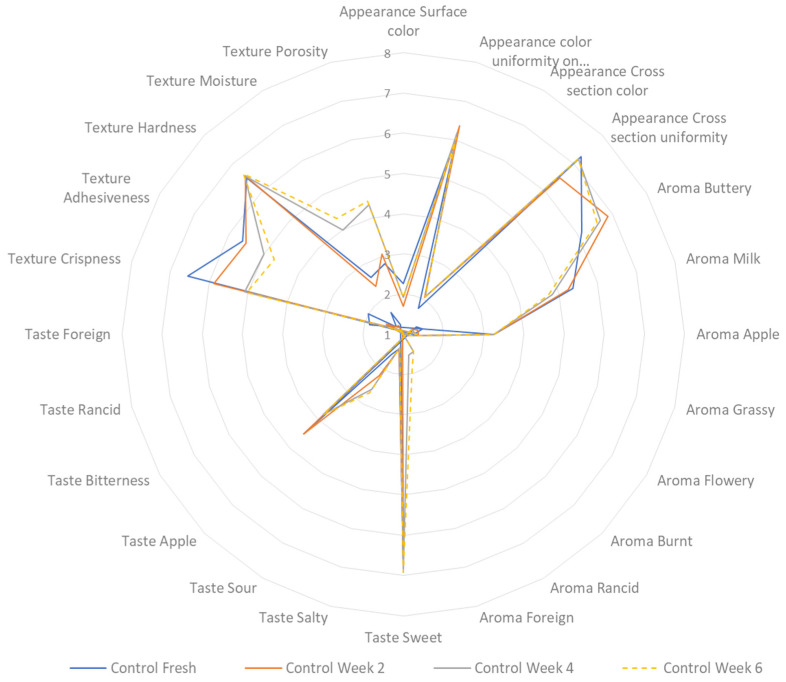
Sensory profiling of control sample during 6 weeks of storage.

**Figure 6:**
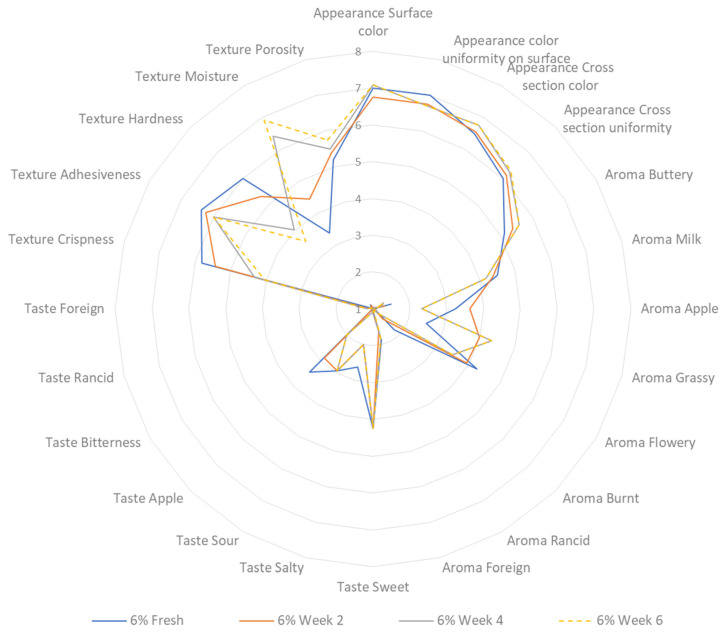
Sensory profiling of sample with 6% CT flower during 6 weeks of storage.

A sensory profiling analysis was carried out on both freshly baked and stored cookies at two-week intervals over a period of six weeks. The cookies were stored at ambient temperature, in tightly closed plastic bags, and shielded from exposure to light. Figure 5 displays the sensory profiling outcome of the control sample, while Figure 6 shows the result for the treated sample. The addition of 6% CT flower had a significant effect on the appearance of the sample. The colour shifted towards a darker shade of blue compared to the control sample, which had a lighter brown colour. This change in colour can be attributed to the process of sugar caramelisation and the Maillard reaction. The Maillard reaction occurs when the free amino group of lysine, peptides, or protein reacts with the carbonyl groups of reducing sugars. This reaction is known to occur during the baking process of bread products, and it speeds up the browning reaction [62,63]. According to the evaluators, there was no significant alteration in the appearance of both samples after storage.

Regarding the aroma, both the control and treated samples exhibit two dominant odours, namely buttery and milky odours. The control sample has a stronger apple aroma, which can be attributed to the higher concentration of apple peel powder (24% of wheat flour *w*/*w*). In contrast, the treatment sample has a more noticeable flowery smell, likely due to the inclusion of flower in the recipe. Both samples exhibit a burnt scent as a result of the baking process, during which sugar undergoes caramelisation [62]. Both samples exhibit prominent flavours of sweetness and apple. Nevertheless, the control sample has a more pronounced apple taste as a result of its higher composition. During the analysis of aroma and taste, it was observed that the rancidity score of the control sample increased significantly compared to the sample containing 6% CT flower. This observation is consistent with the results of the lipid peroxide value, which indicated that the control sample had undergone oxidation at a higher rate than the treated sample. There is no significant difference in the foreign aroma and flavour across the samples, both initially and after storage.

After conducting texture profiling, the control sample exhibits a better score for crispness compared to the treated sample. There is no noticeable difference in the adhesiveness of the samples. The control sample exhibited a higher level of hardness compared to the treated sample. The moisture content in the treated sample did not show a significant difference compared to the control. However, the storage duration had an impact on the moisture content in both samples, showing an increasing trend. In addition, the panellists determined that the storage duration had an impact on the porosity of the control sample, with an increased porosity compared to the treated sample.

## 4. Conclusions

The experiment was performed to improve the traditional recipe of cookies by adding CT flower at concentrations ranging from 0 to 8%. Based on the sensory profiling analysis, a concentration of 6% was selected for future analysis since it exhibited the most desirable organoleptic attributes. The chemical composition analyses revealed that the addition of 6% (*w*/*w* wheat flour) CT did not have a significant impact on the protein, lipid, and ash content of the cookies. However, it did decrease the energy value and significantly increased the levels of insoluble and soluble fibre. The inclusion of 6% CT flower in the cookies had a significant effect on the overall total phenolic content (TPC), which increased compared to the control sample. The findings from the antioxidative activity analyses demonstrate that the inclusion of 6% CT flower resulted in enhanced antioxidative activity in the ABTS, DPPH, ORAC, and PCL assays. There are notable variations observed in the ABTS, DPPH, and ORAC assay results after 6 weeks of storage, indicating a consistent decrease with time. Nevertheless, the PCL-ACL assay did not show any substantial variation, although it did suggest a downward trend. The inclusion of 6% CT flower had a substantial inhibitory effect on the production of hydroperoxide concentration in cookies. Over a period of 6 weeks, a significant rise in peroxide value was observed during the 4th and 6th week of storing enriched cookies. In contrast, the increase in peroxide value in the control sample was first observed during the 2nd week. All assessed products met the high microbiological quality standards. The sensory evaluation scores demonstrate that it is feasible to create cookies with several health benefits and a good overall acceptance score through proper formulation. During the process of storage, the intensity of the buttery aroma increases while the aroma and taste of apple diminish, and the texture gradually becomes softer. Nevertheless, there are no significant changes in the visual appearance. According to the results obtained, the CT flower can be extensively utilised in baked cookies as a rich source of polyphenols with strong antioxidant properties and high fibre content. This can potentially offer many advantages for human health and overall well-being.

## Figures and Tables

**Table 1:** The formulation of cookies.

Ingredients [g]	Control Sample	Dried CT Flower Powder Concentration			
		2%	4%	6%	8%
Wheat flour	38.00	37.24	36.48	35.72	34.96
Apple peel powder	12.00	11.76	11.52	11.28	11.04
CT flower powder	0.00	1.00	2.00	3.00	4.00
Coconut oil	10.00	10.00	10.00	10.00	10.00
Butter	15.00	15.00	15.00	15.00	15.00
Sweetened condensed milk	25.00	25.00	25.00	25.00	25.00
Salt	0.50	0.50	0.50	0.50	0.50
Total	100.50	100.50	100.50	100.50	100.50

**Table 2:** Colour characteristics of cookies with CT.

Treatment	L*	a*	b*	E
Control	46.12 ± 1.35 a	15.35 ± 0.30 a	32.08 ± 0.93 a	58.24 ± 1.65 a
2%	32.61 ± 0.57 bc	10.74 ± 0.11 b	22.31 ± 0.40 b	40.94 ± 0.64 b
4%	34.79 ± 1.06 b	1.99 ± 0.14 c	12.61 ± 0.30 c	37.06 ± 1.09 c
6%	33.50 ± 0.16 b	0.40 ± 0.05 d	8.44 ± 0.11 d	34.55 ± 0.17 c
8%	30.43 ± 0.47 c	0.94 ± 0.11 e	8.87 ± 0.02 d	31.71 ± 0.45 d

a, b, c, d, e—means in a column followed by the same small letter are not significantly different (*p* > 0.05); values are means of three determinations ± SD.

**Table 3:** Basic composition of cookies with CT.

Nutrient		Sample	
		0%	6%
Protein	[%]	8.41 ± 0.31 a	8.40 ± 0.32 a
Lipid		27.71 ± 0.67 a	27.46 ± 1.30 a
Carbohydrate		57.58 ± 0.39 a	57.29 ± 1.40 a
Ash		1.39 ± 0.05 a	1.26 ± 0.07 a
Moisture		4.91 ± 0.03 a	5.59 ± 0.07 b
Energy	[kcal/100 g]	513.38	509.90

a, b—means in a row followed by the same small letter are not significantly different (*p* > 0.05); values are means of three determinations ± SD.

**Table 4:** Dietary fibre and its fractions in fresh cookies with CT.

Dietary Fibre (%)	Sample	
	0%	6%
IDF	8.41 ± 0.44 b	9.20 ± 0.52 a
SDF	6.40 ± 0.41 b	9.62 ± 0.49 a
TDF	14.82 ± 0.39 b	18.82 ± 0.03 a

a, b—means in a row followed by the same small letter are not significantly different (*p* > 0.05); values are means of three determinations ± SD.

**Table 5:** Pearson’s correlation test coefficient between total phenolics content and antioxidative activities and lipid peroxide value of cookies with CT.

Samples	TPC with	Pearson’s r	Sig. (2-Tailed)
Control	ABTS	0.915	*p* < 0.01
	DPPH	0.935	*p* < 0.01
	ORAC	0.715	*p* < 0.01
	PCL-ACL	0.854	*p* < 0.01
	PCL-ACW	0.472	*p* < 0.05
	LPV	−0.863	*p* < 0.01
6%	ABTS	0.884	*p* < 0.01
	DPPH	0.694	*p* < 0.05
	ORAC	0.790	*p* < 0.01
	PCL-ACL	0.777	*p* < 0.05
	PCL-ACW	0.755	*p* < 0.05
	LPV	−0.910	*p* < 0.01

**Table 6:** Lipid peroxide value of cookies with CT.

**Peroxide value** **(meq O_2_/kg)**	**Storage time (week)**	**Sample**	
		**0%**	**6%**
	0	20.63 ± 0.15 aB	18.34 ± 0.02 aA
	2	21.64 ± 0.14 abB	18.52 ± 0.64 aA
	4	22.70 ± 0.82 bcB	19.89 ± 0.01 abA
	6	24.28 ± 0.45 cB	21.55 ± 0.96 bA

a, b, c—means in a column followed by the same small letter are not significantly different in each analysed storage time (*p* > 0.05); A, B—means in a row followed by the same capital letter are not significantly different in each analysed sample (*p* > 0.05); values are means of three determinations ± SD.

**Table 7:** Microbiological identification of cookies with CT.

	Aerobic Mesophilic Microorganisms Count [cfu/g]	Aerobic Psychrophilic Microorganisms Count [cfu/g]	Yeasts and Moulds Count [cfu/g]	*Enterobacteriaceae* Count [cfu/g]	Presence of *Staphylococcus* spp.	Presence of *Salmonella* sp.
C0	ABS 0.1 g	ABS 0.1 g	ABS 0.1 g	ABS 0.1 g	ABS 0.1 g	ABS 25 g
C6	ABS 0.1 g	ABS 0.1 g	ABS 0.1 g	ABS 0.1 g	ABS 0.1 g	ABS 25 g
S0	ABS 0.1 g	ABS 0.1 g	ABS 0.1 g	ABS 0.1 g	ABS 0.1 g	ABS 25 g
S6	ABS 0.1 g	ABS 0.1 g	ABS 0.1 g	ABS 0.1 g	ABS 0.1 g	ABS 25 g
CT	ABS 0.1 g	ABS 0.1 g	ABS 0.1 g	ABS 0.1 g	ABS 0.1 g	ABS approx. 20 g
AP	ABS 0.1 g	ABS 0.1 g	ABS 0.1 g	ABS 0.1 g	ABS 0.1 g	ABS approx. 20 g

C0—control cookies week 0, C6—control cookies week 6, S0—cookies 6% week 0, S6—cookies 6% week 6, CT—*Clitoria ternatea* powder, AP—apple peels freeze-dried, ABS—absence in.

## Data Availability

The data presented in this study are available on request from the corresponding author (RRM, and AGM).
